# Effect of CMNa combined with radiotherapy on the tumor immune microenvironment of mouse cervical cancer cell transplantation tumor model

**DOI:** 10.1080/21655979.2021.1899532

**Published:** 2021-03-30

**Authors:** Li Li, Weiqiang Shi, Juying Zhou

**Affiliations:** a Department of Radiation Oncology, The First Affiliated Hospital of Soochow University, Suzhou, China; b Department of Pathology, The First Affiliated Hospital of Soochow University, Suzhou, China

**Keywords:** Forkhead transcription factor (Foxp3), T cell activation inhibitor immunoglobulin variable domain (VISTA), cervical cancer, immune microenvironment, glycididazole sodium (CMNa)

## Abstract

In this study, we construct a subcutaneous tumor mice model of U14 cells, observe the tumor growth, and detect the expression of Foxp3 and VISTA in cervical cancer tissues and adjacent tissues during CMNa-enhancing radiotherapy.From the 15th day, compared with the control group, the tumor volume changes in each treatment group were significant (P < 0.01). CMNa combined with radiotherapy had an interactive effect and a positive effect in inhibiting tumor volume growth. There was no significant difference in the expression of Foxp3 and VISTA in mouse cervical cancer tissues and adjacent tissues in each group. The Foxp3 level in the RT group was the highest, and the CMNa group was the lowest. The VISTA level of the CMNa+RT group was the highest, the RT group is followed by, and the Control group is the lowest. The Foxp3 level of the CMNa group did not change much at each different point. The Foxp3 level in RT and CMNa+RT group gradually decreased after a transient increase, and the VISTA level in the CMNa+RT group increased more.Our results show that CMNa can enhance the efficacy of radiotherapy, and at the same time can reduce the compensatory increase in regulatory T cell Foxp3 levels caused by radiotherapy, and reduce the radiotherapy response. However, in the course of the treatment of the two, there may be a substantial increase in the level of VISTA, and the combined application of VISTA inhibitors may increase the anti-tumor response.

## 1. Introduction

Cervical cancer is a common gynecological malignant tumor in the world, especially in developing countries, and its incidence is the fourth largest among female tumors [1]. The treatment of advanced cervical cancer mainly includes radiotherapy and chemotherapy. With the advancement of radiotherapy technology and the emergence of new chemotherapeutic drugs, the curative effect is correspondingly improved [2]. However, the overall 5-year survival rate is still not high. Orbegoso et al. reported that 40% of advanced cervical patients quickly developed disease [3]. Analysis of the occurrence of cervical cancer is mostly related to high-risk HPV virus infection. When the body’s immunosuppressive effect cannot effectively eliminate the virus, the local cervical immune microenvironment is unbalanced, which in turn leads to the occurrence and development of cervical cancer. Researchers have observed that the expression of negative immune checkpoint PD-L1 on the cell membrane is up-regulated in human HPV virus-related tumors [4,5]. Although cervical cancer cannot yet be classified as an immunogenic tumor, more and more evidences show that cervical cancer has an endogenous anti-tumor immune response, which indicates the feasibility of immunotherapy in cervical cancer research.

In recent years, major breakthroughs have been made in immunotherapy. However, the tumor microenvironment plays an important role in suppressing or enhancing the immune response. Among them, the forkhead transcription factor (Foxp3) is a specific marker of T-regulatory cells (Treg) [6,7]. When Foxp3 is over-expressed in the local tumor microenvironment, it helps tumor cells escape the surveillance of the immune system and promotes tumor growth. T cell activation inhibitor immunoglobulin variable domain domain (VISTA) is a negative immune checkpoint molecule that inhibits T cell response. It has dual functions of receptor and ligand, and its expression is increased in tumor microenvironment and tumor infiltrating lymph nodes [8]. Ledi et al. [9] found that the high expression of VISTA on immune cells (IC) and vascular endothelial cells (VEC) in tumors is closely related to the stage of advanced cervical cancer and lymph node metastasis (LNM). VISTA has a certain correlation with the prognosis of cervical cancer, and it has the potential as a treatment target.

Glymidazole sodium for injection (CMNa) is a highly effective and low-toxic hypoxic cell sensitizer that is currently officially used in clinical practice. Previous studies have proved [10,11] that in the treatment of cervical cancer, CMNa can improve the tumor control rate without increasing the adverse reactions of radiotherapy.

To study the changes in the tumor immune microenvironment after radiotherapy combined with CMNa sensitization therapy, and to explore the feasibility of radiotherapy combined with immune checkpoint inhibitor therapy. In this study, C57bl/6 mice bearing U14 cells were used as the research object to observe the changes in the expression levels of Foxp3 and VISTA at immune checkpoints before and after radiotherapy and during CMNa-enhancing radiotherapy. We envision that immune checkpoint inhibitor immune targeted therapy, CMNa and radiotherapy can be combined in the treatment of cervical cancer. It is hoped to lay a theoretical foundation for the combination of radiotherapy and immunotherapy for cervical cancer. The combination of immunotherapy and radiotherapy and radiotherapy sensitizers will assist each other.

## 2. Experiment materials and methods

### 2.1 Preparation and feeding of animals

Fifty C57bl/6 mice, female, SPF grade, 6–8 weeks old, weighing 16–22 g, were purchased from the Animal Center of Soochow University and raised in the animal room of Soochow University. The mice were purchased about 2 weeks before the experiment and allowed to adapt to the new environment.

### 2.2 Cell lines

Purchase mouse cervical cancer cells (U14) from the Cell Bank of the Chinese Academy of Sciences. Mouse U14 tumor is a squamous cell carcinoma. In 1958, the experimental tumor group of the Department of Pathology, Institute of Basic Medical Sciences, Chinese Academy of Medical Sciences used 20-methylcholanthrene to thread and induce ectopic cervical cancer. The strain was established and stored. The initial pathological structure was similar to carcinosarcoma, and it was clearly undifferentiated after the 1980s. The average survival time of tumor-bearing mice was 27 days. The lymph node metastasis rate is 95%, and the lung metastasis rate is 80%. It is a bidirectional metastatic strain.

### 2.3 Main instruments, medicines and reagents

The optical microscope was produced by Olympus, Japan, and the radiotherapy machine was the Varian 23EX linear accelerator in the Radiotherapy Center of the First Affiliated Hospital of Soochow University. CMNa is a gift from Luye Pharmaceutical Company, Foxp3 polyclonal antibody is produced by Abcam, and VISTA polyclonal antibody is produced by CST. Immunochromogenic reagents are provided by Gene Technology.

### 2.4 Construction of mouse subcutaneous tumor model

Take the U14 cells in the logarithmic growth phase, add physiological saline to dilute them, make them into a cell suspension, count them by the above method, and adjust the cell suspension concentration to 5 × 106/ml for later use. The skin of the left groin of the mouse was disinfected with iodophor. A 1 ml empty needle was used to inoculate 0.2 ml of cell suspension 0.5 cm subcutaneously on the groin of the left lower limb of the mouse. Make it partly present skin hills the size of soybean grains, and observe whether there is any liquid overflow. After inoculation, observe no abnormalities and send them back to the original place to continue feeding. Tumors formed about 1 week after the inoculation. After the soy nodules can be palpated, the growth status of the transplanted tumors was checked the next day and the behavioral changes of the mice were observed. Including mental state, weight, eating and drinking, activities, using vernier calipers to measure the length and short diameter of the transplanted tumor and calculate the tumor volume, and record it completely.

### 2.5 Grouping and treatment of tumor-bearing mice

Make 100 tumor-bearing mice. When the diameter of the transplanted tumor reaches 8–10 mm, the 100 mice are divided into 4 groups according to the random number method, each with 25 mice. After grouping, the quality of the mice in each group was compared, and there was no significant statistical difference (P > 0.05), which proved that the general conditions of the mice in each group were similar before the experiment, indicating that the comparability was better.

Control group (Control group): 0.2 ml physiological saline was injected intraperitoneally. At the same time.

Radiotherapy alone group (RT group): 0.2 ml of normal saline was injected intraperitoneally. The hind limbs of the mice were extended and extended as far as possible, and the trunk and extremities were fixed with tape. Adjust the light field of the accelerator so that the tumor tissue is located in the irradiation area, and try to avoid exposure to the trunk and limbs other than the tumor. The tumor area of the forelimb was locally irradiated with 6Me V-electron line source skin distance, SSD = 100 cm, depth (d) = 1 cm, plus 0.5 cm tissue compensation, dose (DT) = 8 Gy.

Glycidazole sodium group (CMNa group): using intraperitoneal injection, inject CMNa, 60 mg/kg/d, diluted with 0.9% normal saline, about 0.2 ml/mouse.

Glycidazole sodium + radiotherapy group (CMNa+RT group): intraperitoneal injection 4 hours before radiotherapy, injection of CMNa, 60 mg/kg/d, diluted with 0.9% normal saline, about 0.2 ml/head. The radiotherapy method is the same as the radiotherapy group.

The mice were sacrificed by cervical dislocation on 1d, 8d, 15d, 22d, and 29d. The tumor was removed, washed with saline and weighed. The tissue sample was placed in an embedding box, fixed with 10% formaldehyde solution, and set aside.

### 2.6 Observe the tumor growth

Observe the growth of each group of tumors, the maximum diameter a mm and the minimum diameter b mm of the transplanted tumor, and calculate the tumor volume (mm3) with the formula V = ab2 /2. And get the average value, and then draw the growth curve of the four groups of mouse tumors. And observe the behavioral changes of mice, including mental state, eating and drinking, and activities.

### 2.7 Immunohistochemical method to detect the expression of Foxp3 and VISTA in cancerous tissues and adjacent tissues

Using immunohistochemistry SP three-step method. Paraffin sections are conventional xylene, gradient ethanol, dewaxed and hydrated, heated with citrate of pH = 6 in boiling state for 20 minutes, then cooled at room temperature; Wash in phosphate-buffered saline (PBS) for 5 min, repeat 3 times; blocker 3% hydrogen peroxide (H2O2) to remove endogenous peroxidase, room temperature for 15 min; Wash with PBS for 5 minutes and repeat 3 times; add blocking solution (normal goat serum) and block at room temperature for 15 minutes; pour out the blocking solution, (do not wash) drop I antibody, overnight at 4°C; Wash with PBS for 5 minutes, repeat 3 times; Add biotin-labeled anti-rat/rabbit IgG II antibody (reagent C) for 20 minutes at room temperature; wash in PBS for 5 minutes, repeat 3 times; Add horseradish peroxidase-labeled streptavidin (reagent D) for 20 minutes at room temperature; wash with PBS for 5 minutes and repeat 3 times; add the color developing agent DAB, observe under light microscope, stop with water after proper color development; Hematoxylin counterstain the cell nucleus, wash with water; mount the slide with dehydrated, transparent, neutral gum.

### 2.8 Determination of positive results

All sections were double-blind and read by two pathologists independently. After immunohistochemical staining, 3 slices were randomly taken from each sample, and the slices were first observed under a low power microscope (X 40). Select the area with the highest density of infiltrating lymphocytes in cancer nests and interstitial tissues, and then switch to a high-power microscope (X 400) to randomly select 5 different fields of view to count positively stained cells. Cell count analysis was performed on tumor cells with positive expression. The result is judged by the semi-quantitative integral evaluation method: Scoring according to the degree of cell coloration, 0 points for no staining, 1 point for light yellow or light black, 2 points for brownish yellow or brown black, and 3 points for brown or black. Score according to the number of stained cells, 1 point for stained cells in a field of view <5%; 2 points for 5%-25%;26%～75% is 3 points; >75% is 4 points. The multiplication of the two scores is the semi-quantitative test score result.

### 2.9 Statistical methods

SPSS 21.0 was used for statistical analysis. The tumor volume and Foxp3 and VISTA levels were expressed as mean ± standard deviation ($\left(\overset{ˉ}{x}\pm s\right)$). The interaction and individual effects of the two treatment methods were analyzed by 2 × 2 factorial design data analysis of variance. The multi-group measurement data method is a one-way analysis of variance. After the differences between the groups are found to be statistically significant, the LSD method is further used for comparison between each two groups. The two groups of measurement data used t test. For all tests, a two-sided P < 0.05 is considered to indicate a statistically significant difference.

## 3. Results

### 3.1 General changes in tumor-bearing mice

C57bl/6 mice formed tumors 7–10 days after inoculation with U14 cells, with a tumor formation rate of 90%. No mice died abnormally during the experiment. The transplanted tumor is round, nodular, and hard in texture. When the size of the transplanted tumor is small, it is easy to peel off, and when it is large, it has adhesion to the surrounding tissues and is not easy to peel off. One week after tumor formation, the mice’s living conditions, activities and food intake did not change significantly. After 3 weeks, the tumor load increased, and the mice gradually became thinner, less active, lethargic, and food intake also decreased. The transplanted tumor grew fastest in the Control group. The mice were sacrificed according to the experimental plan, and the tumor was taken out. The surface of the tumor was uneven. The tumor was light red, and the inside of the incision was grayish white.

### 3.2 Comparison of tumor volume of mouse cervical cancer transplantation

**Figure 1.**
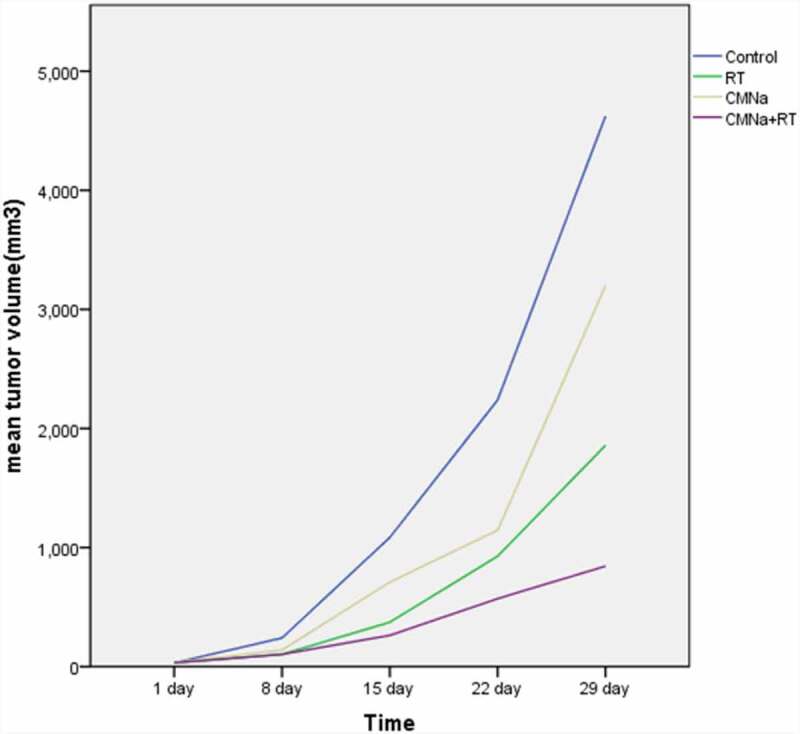
The growth curve of cervical cancer transplanted tumors in each group of mice

The changes of transplanted tumors in each group of mice are shown in (Table 1), and the growth curve is shown in (Figure 1). The tumors in the Control group, RT group, CMNa group and CMNa+RT group all gradually increased at the beginning of treatment. However, the growth rate of the Control group was faster than that of the three treatment groups at all-time points. Statistics found that the tumor volume changes in each treatment group and the Control group from the 15th day were significant (P < 0.01); the inhibitory effect of CMNa+RT group on tumor growth was significantly stronger than that of RT group (P < 0.01) and CMNa group (P < 0.05).

**Table 1.** Comparison of tumor volume of transplanted cervical cancer in mice (mm3, $\left(\overset{ˉ}{x}\pm s\right)$)

Group	1 day after treatment	8 days after treatment	15 days after treatment		22 days after treatment	29 days after treatment
Control	31.10 ± 11.42	241.10 ± 53.07		1085.00 ± 249.69	2240.10 ± 429.93	4622.60 ± 466.20
RT	31.10 ± 11.42	104.00 ± 27.77		373.10 ± 81.49a	927.80 ± 150.67a	1858.40 ± 420.15ac
CMNa	35.50 ± 9.87	139.50 ± 40.59		709.90 ± 208.10a	1146.20 ± 206.36a	3197.70 ± 514.00a
CMNa+RT	32.70 ± 13.17	103.40 ± 17.46a		262.50 ± 48.97ac	571.70 ± 117.16abc	844.00 ± 58.67abc

a. Compared with the Control group, P < 0.05; b. Compared with the RT group, P < 0.05; c. Compared with the CMNa group, P < 0.05.

The results of the analysis of variance with 2 × 2 factorial design showed that CMNa combined with radiotherapy has an interactive effect (F = 5.49, P = 0.0381) and a positive effect in inhibiting tumor volume growth. There is a synergistic effect between CMNa and radiotherapy. When fixed or not using radiotherapy, the tumor volume with CMNa was less than that without CMNa (P < 0.05); When CMNa was fixed with or without CMNa, the size of transplanted tumor was reduced with radiotherapy compared with without radiotherapy, and the difference was statistically significant (P < 0.05).

**Figure:**
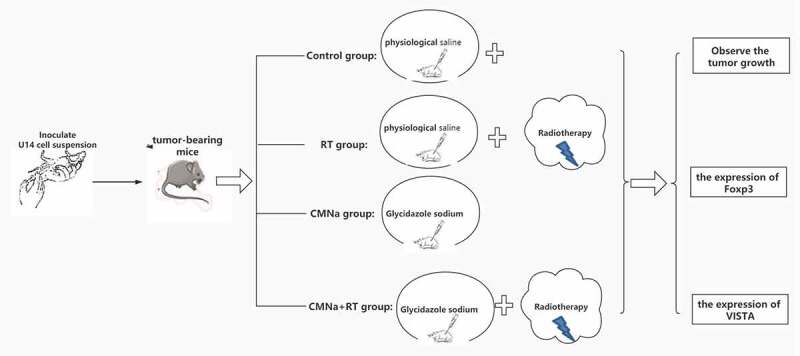

### 3.3 Comparison of Foxp3 and VISTA expression in C57bl/6 mouse tumor tissue and adjacent tissues of each group

**Figure 2.**
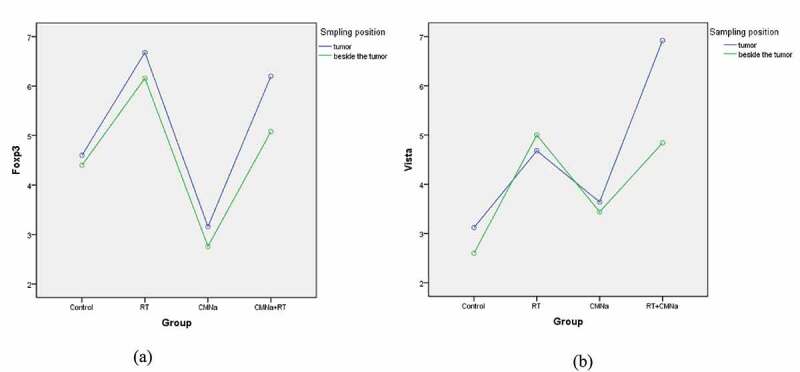
Differences in the expression of Foxp3 and VISTA tumor tissues and adjacent tumors in different groups

As shown in (Figure 2), the expressions of Foxp3 (A) and VISTA (B) in mouse cervical cancer tissues and adjacent tissues were not significantly different in each group.

As shown in (Table 2), in cervical cancer tumor tissue, the levels of Foxp3 and VISTA in the four groups are significantly different. The Foxp3 level in the RT group was the highest, and the CMNa group was the lowest. The comparison between the two groups was statistically significant (P = 0.000). The Foxp3 level in the CMNa+RT group was slightly lower than that in the RT group, but there was no significant difference (P = 0.535), indicating that CMNa can down-regulate the Foxp3 level. The VISTA level of the CMNa+radiotherapy group was the highest, and the difference was statistically significant compared with the other three groups (P = 0.000), followed by the radiotherapy group. The difference between the radiotherapy group, the CMNa group and the Control group was statistically significant (P = 0.029 and 0.001), the CMNa group was slightly higher than the Control group, and the difference was not statistically significant (P = 0.272). It shows that the expression of VISTA increases after radiotherapy, CMNa alone has no effect on the expression of VISTA, but the expression of VISTA can be further increased after combined radiotherapy.

**Table 2.** Comparison of Foxp3 and VISTA levels in mouse cervical cancer tissues (mm3, $\left(\overset{ˉ}{x}\pm s\right)$)

Group	Foxp3	F	P	VISTA	F	P
Control	4.64 ± 2.271	8.602	0.000	4.64 ± 2.271	25.596	0.000
RT	6.68 ± 4.289			6.68 ± 4.289		
CMNa	3.16 ± 1.106			3.16 ± 1.106		
CMNa+RT	6.2 ± 2.217			6.2 ± 2.217		

### 3.4 The changes of Foxp3 and VISTA in C57bl/6 mouse tumor tissues at different times in each group

**Figure 3.**
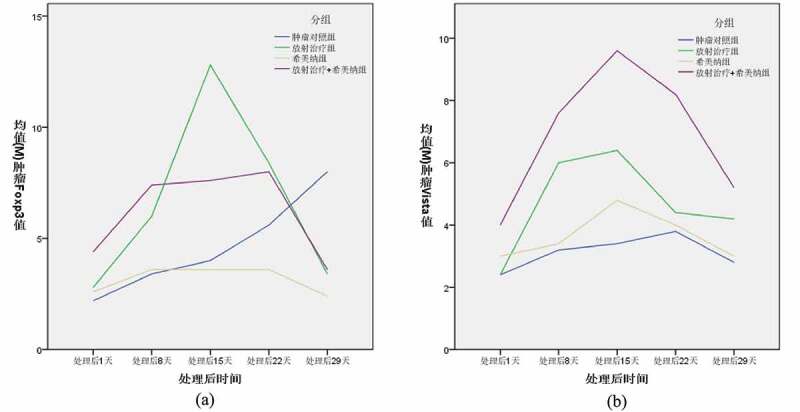
The changes of Foxp3 and VISTA in C57bl/6 mouse tumor tissues in each group at different times

As shown in (Figure 3(a)), the Foxp3 expression levels in the tumor tissues of C57bl/6 mice in each group changed as follows, and there was no significant difference between the groups one day after radiotherapy; The increase in the RT group and CMNa+RT group was 8 days after radiotherapy, which was significantly different from the Control group. 15 days after radiotherapy, the RT group further increased, the CMNa+RT group changed little compared with the previous, the difference between the two groups was significant, and the Control group gradually increased; The decrease in the RT group and the CMNa+RT group at 22 days after radiotherapy was not significant compared with the Control group, and there was a difference compared with the CMNa group; the Control group was higher than the other three groups at 29 days after radiotherapy, and the difference was significant. The Foxp3 level in the CMNa group did not change much at each time point, while the RT group and the CMNa+RT group gradually decreased after a transient increase, and the RT group increased more rapidly, as shown in (Table 3).

**Table 3.** Comparison of Foxp3 levels in mouse cervical cancer tissues at different times (mm3, $\left(\overset{ˉ}{x}\pm s\right)$)

Group	1 day after treatment	8 days after treatment	15 days after treatment		22 days after treatment	29 days after treatment
Control	2.2 ± 0.447	3.4 ± 0.548		4.0 ± 1.000	5.6 ± 1.673	8.0 ± 1.225
RT	2.8 ± 0.837	6 ± 1.414ac		12.8 ± 2.280ac	8.4 ± 4.336 c	3.4 ± 0.548a
CMNa	2.6 ± 0.548	3.6 ± 1.517		3.6 ± 1.140	3.6 ± 1.140	2.4 ± 0.548a
CMNa+RT	4.4 ± 0.894	7.4 ± 1.342ac		7.6 ± 1.517abc	8.0 ± 1.225 c	3.6 ± 1.517a

a. Compared with the Control group, P < 0.05; b. Compared with the RT group, P < 0.05; c. Compared with the CMNa group, P < 0.05.

As shown in (Figure 3(b)), the expression of VISTA in the tumor tissues of C57bl/6 mice in each group had no significant difference 1 day after radiotherapy. 8 and 15 days after radiotherapy, the RT group and the CMNa+RT group increased progressively, which was significantly different from the Control group and the CMNa group. The post-RT group and CMNa+RT group gradually decreased, and the CMNa+RT group was higher than the other three groups at 22 days after radiotherapy. The difference was significant, and there was no significant difference among the other three groups. There was no significant difference between the groups 29 days after radiotherapy. The expression of VISTA in the CMNa group did not change much at each time point, while the RT group and the CMNa+RT group gradually increased after a transient increase, and the increase in the CMNa+RT group was higher, as shown in (Table 4).

**Table 4.** Comparison of VISTA levels in mouse cervical cancer tissues at different times (mm3, $\left(\overset{ˉ}{x}\pm s\right)$)

Group	1 day after treatment	8 days after treatment	15 days after treatment		22 days after treatment	29 days after treatment
Control	2.4 ± 0.894	3.2 ± 0.837		3.4 ± 0.894	3.8 ± 1.483	2.8 ± 0.837
RT	2.4 ± 0.548	6.0 ± 0.000ac		6.4 ± 0.894ac	4.4 ± 0.894	4.2 ± 1.095
CMNa	3.0 ± 0.707	3.4 ± 0.548		4.8 ± 1.095	4.0 ± 1.225	3.0 ± 0.707
CMNa+RT	4.0 ± 0.994	7.6 ± 1.517ac		9.6 ± 1.342ac	9.6 ± 1.342abc	5.2 ± 0.095

a. Compared with the Control group, P < 0.05; b. Compared with the RT group, P < 0.05; c. Compared with the CMNa group, P < 0.05.

## Discuss

This experiment established a mouse cervical cancer model to detect the levels of Foxp3 and VISTA, a T cell activation inhibitor, after CMNa sensitized radiotherapy. To explore the feasibility of combination of CMNa sensitized radiotherapy and immune checkpoint VISTA inhibitors.

The immune system is dually regulated by costimulatory signal molecules and negative immune checkpoint molecules. Among them, negative immune checkpoint molecules can inhibit the activity of T cells to kill tumor cells, blocking these inhibitory signals can improve anti-cancer immunity, and ultimately achieve the goal of eliminating tumors [12,13]. Researchers analyzed the relationship between the status of HPV virus in head and neck squamous cell carcinoma and anal cancer related to human HPV virus and the expression of negative immune checkpoint PD-L1. It has been observed that the expression of PD-L1 on the cell membrane is up-regulated [4,5]. The occurrence of cervical cancer is mostly related to HPV virus infection. When the body’s immunosuppressive effect is strong, the virus cannot be effectively eliminated, and the local immune microenvironment of the cervix is imbalanced, which in turn leads to the occurrence and development of cervical cancer.

VISTA, similar to the B7 Ig superfamily including PD-L1 [14]. In vitro and in vivo, VISTA exerts immunosuppressive activity on T cells, and may be an important mediator to control the development of autoimmunity and the immune response to cancer. Le Mercier et al. [15] proved that the expression of VISTA is increased in the tumor microenvironment and tumor infiltrating lymph nodes. Blocking the expression of VISTA can inhibit the differentiation of natural regulatory T cells and tumor-specific inducible T cells, and hinder tumor growth. Studies have found that the overexpression of VISTA on tumor cells in murine cancer models can trigger the immune protection of tumor cell growth, and the application of VISTA monoclonal antibody treatment can control tumor growth, and the combined use of tumor vaccines will produce significant results [16].

Regulatory T cells (Treg cells) can inhibit the immune response of the body to make the body develop immune tolerance to tumor cells, thereby causing the immune escape of cancer cells. Treg cells mainly express forkhead transcription factor (Foxp3) protein. In some tumors, Foxp3 is related to the activation of CD4 + CD25 + Treg cells, and also affects the development and function of Treg cells, thereby affecting tumor proliferation [17]. In addition, experiments found that in cervical cancer, the high expression of Foxp3 is not only related to poor prognosis, but also an independent prognostic factor predicting overall survival and disease-free survival. The experimental results of Shimizu et al. [18] showed that the number of Foxp3+ Treg infiltration in tumors is related to recurrence and survival.

As early as 1953, studies have found that radiotherapy can cause the reduction or regression of tumor tissue outside the irradiation field. This phenomenon is the abscopal effect of radiotherapy [19]. Up to now, the occurrence of distant effects has only been seen in case reports including melanoma, non-small cell lung cancer, liver cancer, etc [20–22]. In recent years, three-dimensional qualitative radiotherapy and other technologies have become more and more widely used. In theory, this single high-dose radiotherapy is easier to induce remote effects, but the actual incidence is still extremely low, suggesting that there are some factors that hinder anti-tumor immune response [23,24].

The body’s immune activity effect surpasses the immunosuppressive effect and occupies a dominant position, and the remote effect is possible. Radiotherapy will cause a series of complex reactions in the body. On the one hand, radiotherapy can induce immunogenic death of tumor cells, expose tumor antigens to become “in situ tumor vaccines”, and induce the production of damage-related molecular patterns (DAMPs) to promote the maturation of dendritic cells (DC). It is manifested as an immune-promoting effect. On the other hand, immunosuppression can also occur in radiotherapy. First, studies have confirmed that increased levels of Tregs in the tumor microenvironment [25–27] are associated with late tumor staging and poor prognosis [28,29]; However, in a mouse model of subcutaneous transplantation tumors on both legs, local radiotherapy can cause increased Treg and CTL infiltration of the tumors on both legs. Our experiment also found that Foxp3 levels in the radiotherapy group increased progressively at 1 day, 1 week, 2 weeks, 3 weeks, and 4 weeks after radiotherapy. Consider the increased reactivity of immunosuppressive cells such as Treg and MDSCs after radiotherapy [30–32]. In addition, studies have found that in PD-1 knockout mouse melanoma and renal cell carcinoma models, local radiotherapy (15 Gy×1 times) can cause PD-L1 expression. Our research also found that the expression of VISTA at the negative immune checkpoint gradually increased after radiotherapy. These are all unfavorable factors affecting tumor immunity. It shows that although radiotherapy can activate tumor-specific immune responses, the immune system will also have negative immune checkpoints and other suppressive factors after radiotherapy. In addition to the tumor microenvironment that is already in a highly immunosuppressive environment, radiotherapy will also hinder the killing effect of cellular immunity on tumor cells [33].

Therefore, we propose that radiotherapy combined with immune checkpoint inhibitors can be used to treat cervical cancer. In mouse breast and colon cancer models, the researchers combined radiotherapy and CTLA-4 inhibitors significantly inhibited lung metastasis and prolonged the overall survival of mice [34–36]. When combined with CTLA-4 inhibitors, the ratio of Treg/CTL within the tumor decreased significantly, and the release of inflammatory factors increased, which eventually caused the inhibition of distant tumor growth [36]. Case reports in clinical practice also confirmed that this combination therapy can cause clinical remote effects [20–22]: A patient with metastatic melanoma was evaluated as disease progression during the administration of ipilimumab (CTLA-4 inhibitor). After radiotherapy for spinal cord metastases, treatment was continued with ipilimumab, and all lesions were found to be significantly reduced. Monitoring the patient’s hematological indicators found that the number of antigen-presenting cells was significantly higher than before, while MDSC was lower than before, suggesting that combination therapy reduces the increase in immunosuppressive cells caused by radiotherapy; On the other hand, after radiotherapy, the expression levels of negative immune checkpoints in the tumor and adjacent to the tumor are significantly higher than before radiotherapy [37], so the efficacy of immune checkpoint inhibitors after radiotherapy can also be improved compared to before radiotherapy. In other words, radiotherapy can restore or improve the response of tumors to immune checkpoint inhibitor therapy, which is more beneficial for the control of residual tumor cells and tumor micrometastasis after radiotherapy. Our study found that the level of VISTA increased significantly after radiotherapy, suggesting that if radiotherapy is combined with immune checkpoint VISTA antibody, it can down-regulate Foxp3 and VISTA levels, reduce the impact of suppressive immunity, and amplify the efficacy of radiotherapy. In addition, the study found that patients with malignant melanoma who were resistant to ipilimumab in the early stage showed high expression of PD-L1 after radiotherapy, and thus benefited from PD-L1 blockers again [38]. A clinical report about radiotherapy combined with PD-1 antibody in the treatment of Kras wild-type non-small cell lung cancer also confirmed that this combination is safe and feasible [39]. Therefore, radiotherapy combined with immunotherapy can not only reduce the influence of suppressive immune factors. Moreover, under the action of radiotherapy, the immune checkpoint is increased, and the corresponding checkpoint inhibitor needle therapy will be further amplified, which is better than single radiotherapy or immunotherapy. With more understanding of the remote effects of radiotherapy and immunotherapy, the use of this combination therapy to treat many malignant tumors becomes more and more feasible.

CMNa is connected to the chemical structure of the pro-tumor cell to capture the electrons on the target molecule damaged by radiotherapy of tumor cells, thereby forming cationic free radicals, accelerating tumor cell death and enhancing the damage effect. And it can inhibit the damaged DNA molecule polymerase B in tumor cells, thereby hindering the repair of DNA, and achieving the purpose of improving the efficacy of radiotherapy. From our experiments, the Foxp3 protein level was the highest in the radiotherapy group, and CMNa down-regulated the Foxp3 protein level. While CMNa sensitized radiotherapy, the negative immune regulation point VISTA in the immune microenvironment was further increased than the radiotherapy group alone. It is speculated that the influence of CMNa on the immune microenvironment has the following aspects: 1. CMNa can significantly improve the immune function of the body. It can directly or indirectly down-regulate the expression of TregFoxp3, reduce the immunosuppression caused by radiotherapy, and improve the microenvironment. Studies have found that in the treatment of cervical cancer, the combined use of CMNa has higher levels of CD3+, CD4+, CD4+/CD8+ (P < 0.05), and lower CD8+ levels (P < 0.05) compared with the radio-chemotherapy group alone. It shows that CMNa can reduce the side effects of radiotherapy by improving the immunosuppressive state in the microenvironment [40]. 2. CMNa combined with radiotherapy and chemotherapy can reduce the level of HIF-1ɑ in patients with cervical cancer. Many studies have confirmed that the silence of HIF-1ɑ expression can significantly inhibit the growth of transplanted tumors [41,42], and significantly down-regulate the expression of VEGF-C, SDFα, IL-8 and G-CSF [43–46]. Therefore, CMNa may inhibit the growth and infiltration of tumor cells by improving hypoxia, reducing the expression of HIF-1α, and reducing the expression of VEGF. 3. CMNa can selectively act on tumor hypoxic cells, but has no obvious activity on normal oxygenated cells. After being combined with tumor hypoxic cells, CMNa affects its repair by competitively binding electrons with the free radicals of biological target molecules induced by radiotherapy. As a result, tumor hypoxic cells are reduced, and the negative immune regulatory point VISTA in the immune microenvironment is further increased. Li Weidong et al. pointed out [47] that hypoxic microenvironment can promote cancer cell metastasis and infiltration, which is one of the key factors leading to treatment tolerance and an important factor for resistance to anti-tumor immune response. Therefore, CMNa sensitization radiotherapy combined with immune checkpoint inhibitor therapy may improve the therapeutic effect.

In summary, our research shows that radiotherapy itself can increase the expression of VISTA and other immunosuppressive signals in tumor cells and tumor microenvironment and induce an increase in the number of Foxp3, and CMNa sensitization radiotherapy combined with immune checkpoint inhibitor therapy can achieve mutual benefits. CMNa can sensitize the efficacy of radiotherapy, and can also reduce the compensatory increase in Foxp3 level caused by radiotherapy, and reduce the radiotherapy response. At the same time, it may cause the VISTA level of immune checkpoint to further increase. If the immune checkpoint VISTA inhibitor is combined at this time, it will improve the anti-tumor efficacy, and this benefit can only occur when the CMNa sensitization radiotherapy is combined with the VISTA inhibitor treatment at the same time. The curative effect is better than the treatment with VISTA inhibitor alone, which will also increase the incidence of distant effects. At present, evidence shows that high-dose fractionated radiotherapy is more closely related to the generation of distant effects. Therefore, research on the optimal dose and treatment method is very important for optimizing radiotherapy combined with immunotherapy, which may be affected by the biological behavior of different tumors and the limitations of experimental animal models. And for the observation of the side effects of the combination therapy, these all need further research and clinical verification.

## Conclusion

CMNa can enhance the efficacy of radiotherapy, and at the same time can reduce the compensatory increase in regulatory T cell Foxp3 levels caused by radiotherapy, thus reducing the occurrence of radiotherapy side effects. However, in the course of the treatment of the two, there may be a substantial increase in the level of VISTA. It mesns that if combined application of VISTA inhibitors may increase the anti-tumor response, and this benefit only occurs when CMNa sensitizing radiotherapy is given in combination with VISTA inhibitor treatment at the same time.

## Data Availability

All data, models, and code generated or used during the study appear in the submitted article.
